# Insulin edema in slowly progressive type 1 diabetes: improvement following adjustment of insulin therapy

**DOI:** 10.1007/s13340-025-00864-4

**Published:** 2025-12-29

**Authors:** Emi Okamura, Norio Harada, Kana Okuno, Kana Yamamoto, Takaaki Murakami, Yohei Ueda, Daisuke Yabe

**Affiliations:** 1 Department of Diabetes, Endocrinology and Nutrition, Kyoto University Graduate School of Medicine, 54 Shogoin-Kawahara-Cho, Sakyo-Ku, Kyoto, 606-8507 Japan; 2 Department of Endocrinology and Metabolism, School of Medical Sciences, University of Fukui, 23-3 Matsuoka Shimoaizuki, Eiheiji-Cho, Yoshida-Gun, Fukui, 910-1193 Japan

**Keywords:** Insulin edema, Slowly progressive type 1 diabetes, Insulin regimen, Dietary salt restriction

## Abstract

Insulin edema is an uncommon complication that typically arises soon after initiating insulin therapy, most often in individuals with newly diagnosed diabetes or poorly controlled hyperglycemia. An old report from a single hospital in Africa showed an incidence of 3.5% among 491 insulin-treated individuals. Although the precise pathophysiology remains uncertain, proposed mechanisms include insulin-induced sodium retention, increased vascular permeability, and dysregulation of the renin–angiotensin–aldosterone system. Insulin edema has been described in both type 1 and type 2 diabetes; however, occurrence in slowly progressive type 1 diabetes mellitus (SPIDDM) is exceptionally rare. We report a woman with SPIDDM who developed bilateral lower-leg edema shortly after starting basal–bolus insulin therapy with insulin aspart and insulin degludec. She exhibited no signs of heart failure, liver disease, renal impairment, or allergic reaction to insulin. Cardiac function was normal on echocardiography, and B-type natriuretic peptide levels were within the reference range. She experienced marked edema and an approximately 7-kg weight gain after insulin initiation. Following modification of the insulin regimen and dietary sodium restriction (8 g/day of salt), the edema resolved rapidly within nine days without the use of diuretics. This case illustrates that insulin edema can occur even in individuals with SPIDDM. The observed improvement after insulin regimen adjustment likely reflects the combined influence of glycemic stabilization, fluid–electrolyte balance, and potential formulation-related factors, rather than a direct causal difference between insulin types. Clinicians should recognize this rare yet clinically important complication and adopt an individualized management approach that includes careful glycemic correction and, when appropriate, adjustment of the insulin regimen.

**Supplementary Information:**

The online version contains supplementary material available at 10.1007/s13340-025-00864-4.

## Introduction

Insulin edema is an uncommon complication that may develop soon after initiating insulin therapy. Since first described by Leifer in 1928, fewer than 100 cases have been documented [1–4]. While the true incidence is unknown, an old report from a single hospital in Africa (1950–1961) showed an incidence of 3.5% among 491 insulin-treated individuals [5]. It is most frequently observed in newly diagnosed diabetes or in individuals with poorly controlled glycemia undergoing rapid correction [2, 3]. Although usually transient, it can occasionally lead to serious complications such as pleural effusion, ascites, or heart failure [6, 7].

The underlying mechanisms are not fully understood. Proposed pathways include renal sodium retention via Na⁺/K⁺-ATPase stimulation, insulin-induced vasodilation and increased capillary permeability mediated by nitric oxide, β-adrenergic stimulation, and endothelial modulation [8, 9]. Chronic hyperglycemia–induced microvascular injury may further promote albumin leakage and interstitial fluid accumulation [8]. Dysregulation of the renin–angiotensin–aldosterone system (RAAS) has also been implicated, supported by reports of improvement with spironolactone [9]. Loop diuretics, particularly furosemide, are often used for symptomatic relief [10–12], and spironolactone may be preferred when RAAS activation is suspected [13]. Conversely, there have been reports of cases in which edema was unresponsive to diuretic therapy but resolved following a reduction in the insulin dose [14]. Resolution of edema has also been reported after switching from insulin glargine to detemir [15] or degludec [16], possibly due to their distinct physicochemical profiles.

Most cases occur in acute-onset type 1 diabetes or poorly controlled type 2 diabetes [2, 3]. Reports involving slowly progressive type 1 diabetes (SPIDDM) are extremely rare, likely due to the gradual β-cell decline and slower progression of hyperglycemia. Here, wereport an unusual SPIDDM case in which insulin edema developed during treatment withinsulin aspart and insulin degludecand resolved after switching toinsulin lispro and insulin glargine

## Case report

The case had undergone a routine medical checkup 16-month earlier, which revealed a body weight of 61.0 kg, a body mass index (BMI) of 24.5 kg/m^2^, a fasting plasma glucose level of 135 mg/dL, and an HbA1c of 6.3%. Approximately five months later, she developed polydipsia, polyuria, and excessive thirst, and lost more than 10 kg over the following six months after the checkup. She subsequently underwent blood testing at a nearby clinic 11 months after the medical checkup, where she was diagnosed with diabetes, with fasting plasma glucose and HbA1c levels of 329 mg/dL and 12.1%, respectively (data on serum C-peptide, body weight and BMI as well as blood and urinary ketone levels at that time were unavailable). She was then started on treatment with vildagliptin, glimepiride, and ipragliflozin. Although these symptoms improved 4 months after the initiation of these drugs, her persistent hyperglycemia prompted further evaluation at another hospital. At that time, her body weight was 45.0 kg (BMI 18.0 kg/m^2^). Laboratory findings revealed a casual plasma glucose level of 314 mg/dL, HbA1c 10.2%, serum C-peptide 0.7 ng/mL, and an anti-GAD antibody titer > 2,000 U/mL, confirming the diagnosis of type 1 diabetes. Urinary ketones were only weakly positive (±), a finding considered attributable to the use of a sodium–glucose cotransporter 2 inhibitor. She was started on basal–bolus insulin therapy consisting of insulin aspart and insulin degludec (insulin degludec, 4 units at bedtime; insulin aspart, 4 units before each meal). Four days later, the doses were increased to degludec 6 units at bedtime and aspart 6 units before each meal. Two weeks after initiation, the degludec dose was further increased to 8 units at bedtime. Although daily weight measurements were not recorded, she developed marked bilateral lower-leg edema and experienced a 7-kg weight gain after insulin initiation, prompting admission to our institution for further evaluation.

On admission, her height was 157.8 cm, weight 49.6 kg, and BMI 19.9 kg/m^2^. Her vital signs were stable: Body temperature 36.6 °C, blood pressure 131/73 mmHg, and heart rate 80 bpm. Physical examination revealed no signs of anemia, lymphadenopathy, or goiter. Chest auscultation was clear bilaterally, with normal heart sounds and no murmurs. The abdomen was soft and non-tender with normal bowel sounds. Neurological examination was unremarkable except for the presence of firm edema in both lower legs. Laboratory findings are summarized in Supplemental Table 1. Mild hypoalbuminemia was present, but no proteinuria was detected (urinary albumin 10.1 mg/day and urinary creatin 0.8 g/day), suggesting a non-renal cause of hypoalbuminemia. Urinary ketones were not detected. Human insulin-specific IgE was negative, excluding an allergic reaction to insulin. Although urinary C-peptide excretion was 38 µg/day, the results of the glucagon stimulation test [plasma glucose (mg/dL): 0 min, 112; 6 min, 150; C-peptide (ng/mL): 0 min, 0.27; 6 min, 0.64] demonstrated severely impaired endogenous insulin secretion, consistent with SPIDDM. Chest radiography showed a cardiothoracic ratio of 43.8%, clear costophrenic angles, and no evidence of pleural effusion or pulmonary congestion. Echocardiography demonstrated a left ventricular end-diastolic diameter of 45.2 mm, end-systolic diameter of 28.8 mm, and ejection fraction of 66.1%, with normal diastolic function (E/A ratio 1.23). The inferior vena cava diameter was 18.9 mm with normal respiratory variability, and there were no signs of volume overload or impaired hemodynamics. Liver enzymes were slightly elevated, but abdominal ultrasound showed no hepatomegaly and preserved hepatic architecture. Serum brain natriuretic peptide was within the normal range, ruling out heart failure.

**Table 1:** Summary of published cases of insulin edema: Clinical characteristics, management and outcomes

Case	Age (years)	Sex	Diabetes type	Duration of diabetes	HbAlcat edema onset	Insulin at edema onset	Insulin changed for edema?	Diuretics used	Dietary salt restriction	Edema type	Clinical course of edema	PMID
	53	F	SPIDDM	Newly diagnosed	10.2%	Degludec/Asp art	Degludec to glargine	No	< 8 g/day salt diet	Peripheral	Resolved after insulin switching	N.A
1	20	M	Type 1	Newly diagnosed	> 14.0	Glargine/Lispro	Glargine to Degludec	No	Not reported	Peripheral	Resolved after switching insulin	38,116,161
2	12	F	Type 1	Newly diagnosed	11.6%	Degludec/Lispro	No	Furosemide	Low salt diet	Peripheral	Responded to furosemide and salt restriction	37,453,114
3	51	F	Type 1	16-year	11.7%	Lispro via pump	No	Hydrochlorothiazide	Not reported	Peripheral	Responded to hydrochlorothiazide	36,180,933
4	46	M	ID DM	N.A	N.A	Detemir/Lispro	Discontinued insulins	No	Not reported	Peripheral	Resolved after stopping insulin	36,017,419
5	14	F	Type 1	3-year	> 14.0%	Glargine/Lispro	No	Furosemide	Salt restriction	Generalized	Responded to furosemide and salt restriction	35,669,309
6	57	M	Type 2	5-year	10.7%	Glargine/Aspart	No	Furosemide	Not reported	Peripheral	Responded to furosemide	35,440,964
7	35	F	Type 2	8-year	12.3%	Glargine	No	Furosemide	Salt restriction	Generalized	Responded to furosemide and salt restriction	34,537,806
8	14	M	Type 1	Newly diagnosed	18.0%	Not reported	No	No	Not reported	Peripheral	Resolved spontaneously	33,549,500
9	63	M	Type 1	30-year	8.3%	Human insulin (U-500 insulin) via pump Aspart to human insulin		Spironolactone	Not reported	Peripheral	Responded to spironolactone	33,178,538
10	40	F	Type 1	Newly diagnosed	13.1%	Glargine/Glulisine	No	Spironolactone	Salt/fluid restriction	Generalized	Responded to spironolactone	32,280,575
11	57	M	Type 2	5-year	19.4%	Degludec/Aspart	Dose reduction	Torsemide	6 g/day salt diet	Peripheral	Responded to insulin dose reduction	28,451,823
12	15	F	Type 1	4-year	Not reported	Human insulin	No	No	Not reported	Generalized	Resolved spontaneously	25,633,550
13	72	M	Type 2	15-year	12.3%	Glargine	Glargine to Detemir	No	Not reported	Peripheral	Resolved after switching insulin	25,563,477
14	39	F	Type 1	11-year	13.0%	Aspart via pump	Dose reduction	Furosemide	Salt/fluid restriction	Generalized	Responded to furosemide	25,282,002
15	20	F	Type 1	5-year	> 13.1%	Not reported (pump)	No	Furosemide	Not reported	Generalized	Responded to furosemide	23,815,567
16	35	F	Type 1	Newly diagnosed	13.3%	Intensive insulin therapy	No	Furosemide	Not reported	Generalized	Responded to furosemide	23,613,606
17	14	F	Type 1	Newly diagnosed	Not reported	Not reported	No	Diuretics	Not reported	Generalized	Resolved spontaneously	23,429,714
18	20	F	Type 1	18-year	18.8%	Glargine/Lispro	No	Furosemide	Not reported	Generalized	Responded to furosemide	23,429,012
19	13	F	Type 1	Newly diagnosed	16.6%	Glargine/Aspart	No	No	Not reported	Peripheral	Resolved spontaneously	23,378,550
20	14	F	Type 1	1-year	16.5%	Human insulin (NPH insulin)	No	No	Not reported	Generalized	Resolved spontaneously	23,094,546
21	14	F	Type 1	Newly diagnosed	12.1%	Human insulin	No	No	Not reported	Peripheral	Resolved spontaneously	21,274,337
22	11	F	Type 1	Newly diagnosed	13.9%	Human insulin	No	No	Not reported	Peripheral	Resolved spontaneously	21,274,337
23	19	F	Type 1	Newly diagnosed	14.0%	Lispro/Detemir	No	No	Salt/fluid restriction	Generalized	Responded to salt/fluid restriction	17,956,453
24	12	F	Type 1	Newly diagnosed	12.0%	Human insulin (NPH insulin)	No	Furosemide	Not reported	Generalized	Responded to furosemide	16,972,977
25	44	M	Type 1	4-month	Not reported	Human insulin (NPH humulin)	No	Spironolactone	Not reported	Generalized	Responded to spironolactone	15,762,045
26	49	F	Type 1	Not reported	Not reported	Human insulin (Mixtard insulin)	No	No	Not reported	Generalized	Resolved spontaneously	10,803,477
27	51	M	Mitochondrial	8-year	15.8%	Human insulin (NPH humulin)	No	No	Not reported	Generalized	Resolved spontaneously	8,591,701
28	31	F	Type 1	14-year	Not reported	Human insulin	No	Ephedrine	Not reported	Generalized	Improved with ephedrine	8,359,096
29	10	M	IDDM	Newly diagnosed	14.5%	Human insulin	No	No	Not reported	Peripheral	Resolved after insulin stopped	7,549,583
30	82	F	Type 2	22-year	Not reported	Lente bovine/porcine insulin	No	Furosemide	Not reported	Generalized	Resolved by bed rest and furosemide	4,014,301
31	35	F	Type 1	Newly diagnosed	Not reported	Human insulin (Actrapid/ Insulatard)	Dose reduction	Furosemide	Not reported	Generalized	Responded to furosemide and salt-poor albumin infusion	3,529,068
32	45	M	Not reported	Not reported	Not reported	Not reported	No	No	Not reported	Generalized	Resolved spontaneously	1,960,163

**Fig. 1:**
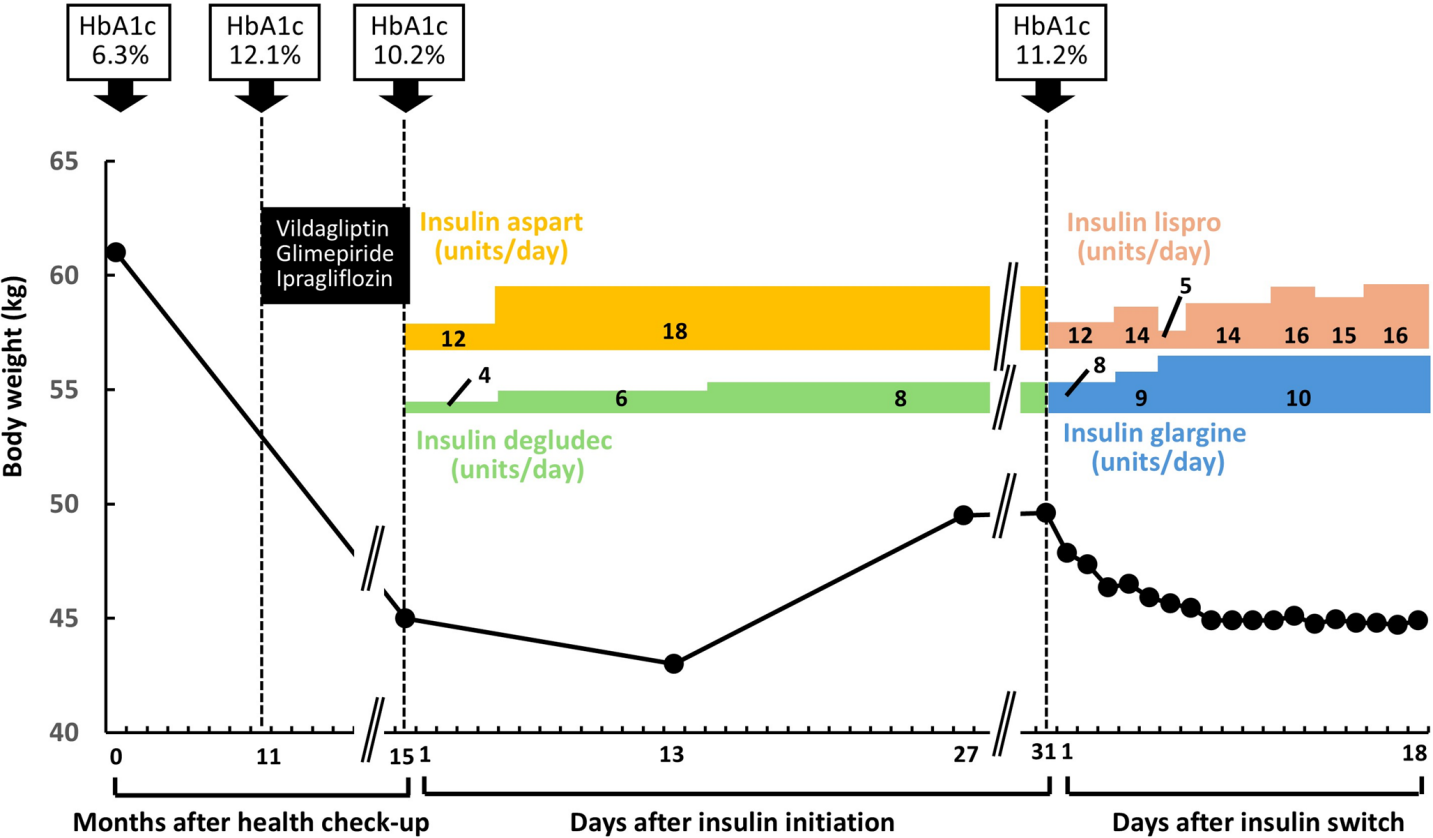
Clinical course and changes in glycemic control, body weight, and insulin therapy in the present case. A 52-year-old woman underwent a routine medical checkup that revealed a body weight of 61.0 kg, fasting plasma glucose of 135 mg/dL, and HbA1c of 6.3%. Five months later, she developed polydipsia, polyuria, and excessive thirst, and lost more than 10 kg over the following six months. Eleven months after the checkup, blood testing at a nearby clinic showed a fasting plasma glucose of 329 mg/dL and HbA1c of 12.1%, leading to a diagnosis of diabetes. She was started on vildagliptin, glimepiride, and ipragliflozin. Although her symptoms improved, persistent hyperglycemia (HbA1c 10.2%) prompted referral to another hospital 15 months later, where her body weight was 45.0 kg, serum C-peptide 0.7 ng/mL, and anti-GAD antibody > 2,000 U/mL, confirming type 1 diabetes. Basal–bolus insulin therapy was initiated with insulin aspart and insulin degludec. Thereafter, she developed marked bilateral lower-leg edema accompanied by a 7-kg weight gain. Upon admission to our hospital, her body weight was 49.6 kg, and HbA1c was 11.2%. Following a switch to insulin lispro and insulin glargine, along with dietary sodium restriction (8 g/day of salt), her edema resolved without the use of diuretics, and body weight decreased to 44.9 kg by day 9

Based on the patient’s clinical course, insulin edema was suspected. The insulin regimen was switched from insulin aspart and insulin degludec to insulin lispro and insulin glargine. The case followed a diet of 1,850 kcal/day with salt adjustment (< 8 g/day), and no diuretics were used. Her weight decreased from 49.6 kg to 44.9 kg by day 9, with resolution of leg edema (Fig. 1). Urinary sodium levels were 3.8 mEq/gCr before the insulin regimen change and 1.9 mEq/gCr seven days afterward. Plasma renin and aldosterone levels were not assessed. Insulin lispro was administered as 7 units before breakfast, 5 units before lunch, and 4 units before dinner; and insulin glargine was given as 10 units before breakfast, successfully maintaining glycemic control.

## Discussion

Insulin edema is an uncommon but well-recognized complication of insulin therapy. Although its underlying mechanisms remain incompletely understood, a combination of insulin-induced sodium retention, altered vascular permeability, and dysregulation of the RAAS is thought to play a role [7, 8]. In approximately 30% of reported cases, insulin edema resolves spontaneously (Table 1); however, when symptoms are severe or persistent, interventions such as dietary sodium restriction and diuretic therapy are often required [5, 6].

IDDM, insulin-dependent diabetes melhtus. A PubMed search using the keywords "insulin", "edema" and "diabetes" identified 1380 articles, from which 32 reports of insulin edema cases documented in English were reviewed in detail and summarized in this table.

As shown in Table 1, insulin edema has been reported with various insulin preparations, suggesting that the phenomenon is not specific to a particular formulation but rather related to the pharmacologic effects of insulin and rapid metabolic shifts during glycemic correction. Nevertheless, differences in formulation excipients, physicochemical properties, or pharmacokinetic profiles may influence the degree of interstitial fluid accumulation in a patient-specific manner, and thus cannot be entirely excluded.

In the present case, edema developed during treatment with insulin degludec and insulin aspart and improved after switching to insulin glargine and insulin lispro. Given that cases of edema have also been reported with glargine [15, 16], the improvement observed here should not be interpreted as evidence of a causal difference between these insulin preparations. Rather, it is likely that multifactorial influences—including adjustment of total insulin dose, rate of glycemic improvement, and concurrent sodium restriction—collectively contributed to the resolution of edema. From a clinical perspective, switching insulin formulations may be considered as one of several empirical management strategies in selected patients with persistent or distressing insulin edema.

Insulin edema is most often reported in individuals with newly diagnosed type 1 diabetes or in those with poorly controlled type 1 and type 2 diabetes who undergo rapid correction of hyperglycemia [2, 3]. Reports in SPIDDM are extremely rare, presumably due to gradual b-cell decline and slower glycemic deterioration. In the present case, although ketosis at the time of diabetes diagnosis was not confirmed, the patient initially responded to oral hypoglycemic agents and did not require insulin therapy for at least four months after diagnosis, despite being positive for anti-GAD antibodies. This clinical course indicates that she was not insulin-dependent at disease onset. However, the fasting C-peptide levels were 0.27 ng/mL during hospitalization at our institution and remained < 0.6 ng/mL (i.e., 0.40 ng/mL) at 18 months after discharge, which is consistent with the diagnostic criteria for SPIDDM. These clinical and biochemical findings—autoantibody positivity, delayed progression to insulin dependence, and eventual marked β-cell failure—collectively provide compelling circumstantial evidence supporting the diagnosis of SPIDDM.

Insulin edema in this case likely developed as a result of relatively rapid correction of long-standing hyperglycemia after initiation of insulin therapy, although the exact glycemic trajectory before admission was unavailable. Regardless of the type of diabetes, including mitochondrial diabetes (Table 1), when edema develops after a rapid improvement in hyperglycemia induced by insulin, insulin edema should be considered in the differential diagnosis.

In conclusion, we describe a rare case of insulin edema in SPIDDM. Although uncommon, this complication can significantly impair quality of life. Clinicians should remain aware of its possibility and consider insulin formulation switching—alongside dietary and pharmacologic measures—as part of an individualized management strategy to optimize outcomes.

## Supplementary Information

Below is the link to the electronic supplementary material.

Supplementary file1 (DOCX 19 kb)

## Data Availability

Clinical data from the corresponding author **is** available upon request.
