# Supplementary material for: Insulin edema in slowly progressive type 1 diabetes: improvement following adjustment of insulin therapy
Source: Diabetol Int. 2025 Dec 29;17(1):13. doi: 10.1007/s13340-025-00864-4 (PMC12748414; doi:10.1007/s13340-025-00864-4)
Supplement: Supplementary file 1 — Supplementary file1 (DOCX 19 kb) [file 13340_2025_864_MOESM1_ESM.docx]

**Supplemental Table 1. Laboratory data upon admission.**

| **Complete blood count** | | | | |  | Amylase | 88 | U/L |
| --- | --- | --- | --- | --- | --- | --- | --- | --- |
| WBC | | 4,350 | | /μL |  | LDL-C | 103 | mg/dL |
| Neu | 64.0 | | % | |  | HDL-C | 108 | mg/dL |
| Lymph | 21.1 | | % | |  | TG | 117 | mg/dL |
| Mono | 7.6 | | % | |  | Glucose | 218 | mg/dL |
| Eos | 6.4 | | % | |  | HbA1c | 11.2 | % |
| RBC | 432×10^４^ | | /μL | |  | Anti-GAD Ab | 2000≧ | U/mL |
| Hb | 13.6 | | g/dL | |  |  |  |  |
| Plt | 31.0×10^4^ | | /μL | |  | TSH | 2.746 | µIU/mL |
| **Biochemistry** | | | | |  | Free T4* | 0.81 | ng/dL |
| AST | | 41 | | IU/L |  | Free T3 | 2.10 | pg/mL |
| ALT | | 102 | | IU/L |  | TgAb | 14 | IU/mL |
| LDH | | 214 | | IU/L |  | TPOAb | 9 | IU/mL |
| ALP | | 214 | | IU/L |  | BNP | 17.3 | pg/mL |
| γGT | | 85 | | U/L |  | IgE | 11 | U/mL |
| TP | | 5.9 | | g/dL |  | Anti-insulin IgE | <0.10 | UA/mL |
| ALB | | 3.8 | | g/dL |  |  |  |  |
| BUN | | 16 | | mg/dL |  |  |  |  |
| Cr | | 0.46 | | mg/dL |  |  |  |  |
| Na | | 142 | | mEq/L |  |  |  |  |
| K | | 4.1 | | mEq/L |  |  |  |  |
| Cl | | 106 | | mEq/L |  |  |  |  |

Ab, antibody; AST, aspartate aminotransferase; ALB, albumin; ALT, alanine aminotransferase; BUN, blood urea nitrogen; Cr, creatinine; Eos, eosinophis; FT3, free triiodothyronine; FT4, free thyroxine; γGT, gamma-glutamyl transferase; HDL-C, high density lipoprotein-cholesterol; LDH, lactate dehydrogenase; LDL-C, low density lipoprotein-cholesterol; Lymph, lymphocytes; Mono, monocytes; Neu, neutrophils; Plt, platelet; RBC, red blood cell; TG, triglycerides; TgAb, anti-thyroglobulin antibody; TPOAb, anti-thyroid peroxidase antibody; TP, total protein; TSH, thyroid-stimulating hormone; WBC, white blood cell.* Between January and August 2023, our facility employed an automated enzyme immunoassay device (AIA-360) for the measurement of thyroid function. It was observed that the results were approximately 0.2 lower compared to the previous method (ECLusys). Considering this, we believe that the fT4 level in this case falls within the reference range.
